# Penetrating Abdominal Trauma: Descriptive Analysis of a Case Series From an Indian Metropolitan City

**DOI:** 10.7759/cureus.32429

**Published:** 2022-12-12

**Authors:** Sumathi Nadikuditi, Nachappa Sivanesan Uthraraj, Vandana Krishnamurthy, Karan Kumar, Meghanaprakash Hiriyur Prakash, Laya Manasa Sriraam, Gokul K Shanker Ramasamy, Kannaki Uthraraj Chettiakkapalayam Venkatachalam

**Affiliations:** 1 General Surgery, University College of Medical Sciences (UCMS) & Guru Teg Bahadur (GTB) Hospital, New Delhi, IND; 2 Trauma and Orthopaedics, William Harvey Hospital, Ashford, GBR; 3 Trauma and Orthopaedics, Jagadguru Jayadeva Murugarajendra (JJM) Medical College and Bapuji Hospital, Davanagere, IND; 4 Research and Development, SIMFFER (Sivameds Fetal Medicine & Fertility Research Foundation) Foundation - Thamarai Health Care, Coimbatore, IND; 5 Anaesthesiology, Safdarjung Hospital, New Delhi, IND; 6 Anaesthesiology, Manipal Hospital, Bengaluru, IND; 7 Cardiology, Banner - University Medical Center Phoenix, Phoenix, USA; 8 Otolaryngology, Kanchi Kamakoti Childrens Hospital, Chennai, IND; 9 General Surgery, VGM Gastro Centre, Coimbatore, IND; 10 Reproductive Genetics, SIMFFER (Sivameds Fetal Medicine & Fertility Research Foundation) Foundation - Thamarai Health Care, Coimbatore, IND; 11 Reproductive Medicine, The Fertility Center, Kovai Medical Center and Hospital (KMCH), Coimbatore, IND

**Keywords:** gun-shot wound, stab injury, abdomen, penetrating injuries, major trauma

## Abstract

Introduction

Penetrating abdominal trauma (PAT) is a major injury that patients present to the emergency department in developed and developing countries. There are many modes and causes of injury. The aim of this study is to analyse the patterns of presentation and parameters at assessment, including investigations, interventions and outcomes of penetrating abdominal trauma at a major trauma centre in an Indian metropolitan city.

Methods

This is an observational descriptive study done over 18 months at a major trauma centre in a metropolitan city in India. The study was registered with the institutional ethics committee and the patients were recruited after obtaining consent on admission. The relevant details were collected from the patient’s electronic records after admission and analysed.

Results

Stab wounds in the 21-40-year-old subset were the commonest. The small intestine was the most commonly injured organ. The mortality rates and the duration of the hospital stay were similar to other case series of the same condition.

Conclusion

The analysis of our case series has highlighted the patterns and outcomes of penetrating abdominal trauma in an urban demographic of a developing economy. Individuals in the prime of their lives, unfortunately, are victims of this mode of injury. Better implementation of standard management protocols can improve outcomes.

## Introduction

Penetrating abdominal trauma (PAT) is an important reason for emergency department attendance, accounting for 9-12% of all trauma presentations [1,2]. In Germany, penetrating trauma contributes to 5% of trauma presentations, including 30% of abdominal penetrating trauma - 36% from gunshot wounds and 12% from stab wounds [3]. In the United States, PAT contributes to 38% of all penetrating traumas [4]. In the UK, there has been an increase in penetrating abdominal trauma over the years, attributable to a 30% increase in firearm and knife crimes [5].

The abdomen includes the peritoneal cavity, the retro-peritoneal space and the pelvis. The intrathoracic part of the abdomen is located within the lower part of the rib cage and extends up to the fourth intercostal space during full expiration [6]. A penetrating abdominal injury is caused due to the mechanical force of a foreign object breaching the skin in the abdominal area and inflicting damage to the structures in its path and resulting in an open wound [7]. Penetrating abdominal injuries are associated with significant morbidity and mortality rates due to injury to vascular structures and vital organs [8-11].

Abdominal penetrating injuries are primarily caused by gunshot wounds, stab injuries or occupational accidents [6,12]. Gunshot wounds are further divided into high and low-velocity injuries based on muzzle velocity [13,14].

The American Association for the Surgery of Trauma’s, Organ Injury Scale (AAST-OIS) has been validated for the liver, kidney and spleen. The scores range from 1 to 5, with 5 representing the most severe injury [15].

There have been scant reports of patient characteristics in penetrating abdominal trauma. The aim of this study is to characterise the cause and mode of injury, the organs involved, the initial assessment, investigations, interventions and the 30-day outcomes from a major trauma centre in a metropolitan city in India, over 18 months.

## Materials and methods

This study was undertaken at a major trauma centre in a metropolitan city over an 18-month period. The study was registered and approved (registration and final submission number: 801/802A) with the institute (University College of Medical Sciences, Delhi) and the institutional ethics committee. Informed consent was obtained from all the participants in the study. This study was designed as a prospective observational study. The inclusion criteria were patients presenting to the emergency department of the Guru Tej Bahadur Hospital (GTB) in New Delhi with penetrating abdominal trauma or those who were referred to the emergency department from other hospitals and health centres, subsequently admitted as inpatients and those who consented to be part of the study. There were no age criteria for inclusion. The exclusion criteria were patients who declined consent, were brought dead to the hospital, had other more severe injuries - thoracic and head injuries that required specialist referral and immediate operative intervention and those who did not have the capacity to consent due to the injury/injuries. The patients underwent initial assessment and resuscitation as per the Advanced Trauma Life Support protocol in the emergency department. The vitals of the patients and the Glasgow Coma Score (GCS) were assessed on admission. The relevant haematological, biochemical, pathological and radiological investigations, including extended Focused Abdominal Sonography for Trauma (eFAST), plain radiographs and computed tomography (CT) were done and triaged for conservative or surgical management. Some patients required intensive care post-operatively. The demographics, patient characteristics and other relevant data were obtained from the electronic health records of the patient. The course in the hospital, with the details of the surgical intervention, the 30-day mortality and morbidity outcomes were recorded. For the statistical analysis, we calculated the mean and standard deviation (SD) of continuous variables. The median and inter-quartile range (IQR) were calculated when there was an asymmetric distribution of the data. For the categorical data, the percentages were calculated and tabulated. Some data could be represented both as categorical and continuous variables and have been represented so - the time delay in presentation to the emergency department and the Glasgow Coma Score (GCS) at presentation. Microsoft Excel software (Microsoft, Redmond, WA) was used for the data analysis.

## Results

There were 41,181 emergency department attendances in the time period of the study with 1864 admissions (4.5%). Seventy-three (73) of the admitted patients had abdominal penetrating trauma and were registered for the study (3.9%). The mean age of the patients was 28.19 years (SD=10.19) and the median was 25 years (IQR=14). The majority of the patients were in the 21-40 years age group (54.8%) (Table 1). Males comprised 91.8% of the cohort. Eighty-four point nine per cent (84.9%) of the injuries were due to assault/violence, 11% of the injuries were non-intentional and 4.1% were self-inflicted (Table 2). On classifying based on the mode of injury, 47.9% were stab injuries, 42.5% were gunshot, 5.5% were accidental sharp object injuries, 2.7% were bull-gore injuries and 1.4% were both gunshot and stab injuries (Table 3). The weapons used to cause trauma were country-made firearms (38.4%), knives (38.4%), bull horns (2.7%), glass bottles (2.7%), ice picks (2.7%), iron bars (2.7%), glass shards (1.4%), iron grills (1.4%), iron rods (1.4%), knives and country-made firearms (1.4%), sharp metallic ends of machinery (1.4%), police rifles (4.1%) and scissors (1.4%) (Table 4). High-velocity injuries were 42.47%, low-velocity injuries were 56.16% of the cohort and 1.37% of them were a combination of the two (Table 5). The mean time interval between injury and presentation to the hospital was 186.86 minutes (SD=310.54). A delay in presentation, defined as a delay of more than 60 minutes, was seen in 53.42% of the patients (Table 6). The mean injury-to-intervention time was 336 minutes (SD= 351 minutes). The mean respiratory rate at presentation was 21.26 breaths/minute (SD=5.94). The mean systolic blood pressure was 110.14 mmHg (SD=30.32). The mean diastolic blood pressure was 68.92 mmHg (SD=19.86). The mean heart rate was 97.56 beats/minute (SD=22.86). The mean oxygen saturation (SpO2) was 92.68 (SD=19.97). The mean Glasgow Coma Score (GCS) was 14.48 (SD=1.96). There was mild derangement (14-15) in 93.15% of the patients and severe derangement (3-8) in 5.3% of the patients (Table 7). There was no abdominal organ evisceration in 80.8% of the patients, bowel only in 1.4%, omentum only in 16.4% and both the omentum and bowel in 1.4% (Table 8). The prevalence of co-morbidities in our cohort was as follows: diabetes (6.8%), asthma (1.4%), hypertension (2.7%), cardiomyopathy (1.4%), intravenous drug abuse (4.1%), psychiatric illness (5.5%) and tuberculosis (1.4%) (Table 9). Eighty-two point one nine per cent (82.19%) of the participants had an eFAST scan done; 63% of them had an anomalous finding on the scan. Fifty-five patients (75.3%) were triaged to have plain radiography after the initial assessment with significant findings in 55% of the patients; 3.6% had cardio-pulmonary angle blunting, 1.8% had pneumothorax, 10.9% had air under the diaphragm and pneumo-haemothorax was seen 3.6% of the patients (Table 10). Ninety point four one per cent (90.41%) of patients had a CT scan performed and the findings were as follows: 12 (27.9%) had lung injuries, 4 (9.3%) had diaphragm injuries, 15 (34.9%) had intra-abdominal solid organ injuries, 17 (39.5%) had a pneumoperitoneum or hollow viscus injuries, 11 (25.6%) had vascular injuries, 5 (11.6%) had bone injuries and 2 (4.7%) had head injuries. Thirty-one point five per cent (31.5%) of the patients were transfused blood products during resuscitation. Peritoneal breach and hence local wound exploration was done in 50.7% of the patients. Forty-eight (48) patients (65.8%) underwent surgical intervention (Table 11). Damage control surgery (early laparotomy) in the first 48 hours was performed in 4 of the 48 patients (8.3%) and definitive procedures in 44 of the 48 patients (91.7%) (Table 12). Five of the patients required a second operation (10.4%). The summary of the organs involved is provided in the table with the highest percentage of patients suffering from abdominal vascular injuries (22; 30.1%) and the small bowel in terms of solid intra-abdominal organs (20.3%) (Table 13). The AAST-OIS grading for organ injury is presented in Table 14). The mortality rate for this series was 6.8%. Fifty point seven per cent (50.7%) of the survivors suffered one or more complications (Table 15). Seventeen point eight zero per cent (17.80%) of the patients required intensive care admission - 7 patients (9.6%) were admitted for 1 day or less, 5 patients (6.8%) were admitted from 1 day to 7 days and one patient (1.4%) for more than 7 days (Table 16). The average hospital stay was 9.75 days (S.D.= 9.16). Surgical site infection was the highest complication at 30.1 % (Table 17). 

**Table 1:** Age distribution of patients N - number of patients

Age range	N (%)
<21 years	24 (32.9)
21-30 years	27 (37.0)
31-40 years	13 (17.8)
41-50 years	4 (5.5)
51-60 years	5 (6.8)

**Table 2:** Cause of injury N - number of patients

Cause of injury	N (%)
Non-intentional	8 (11.0)
Self-inflicted	3 (4.1)
Assault/violence	62 (84.9)

**Table 3:** Mode of injury N - number of patients

Mode of injury	N (%)
Gunshot	31 (42.5)
Stab	35 (47.9)
Bull horn	2 (2.7)
Gunshot and stab wound	1 (1.4)
Accidental fall onto a sharp object	4 (5.5)

**Table 4:** Objects causing injury N - number of patients

Object	N (%)
Countrymade firearm	28 (38.4)
Knife	28 (38.4)
Bull horn	2 (2.7)
Glass bottle	2 (2.7)
Ice pick	2 (2.7)
Glass shard	1 (1.4)
Iron bar	2 (2.7)
Iron grill	1 (1.4)
Iron rod	1 (1.4)
Knife and country-made firearm	1 (1.4)
Sharp metallic end of machinery	1 (1.4)
Police rifle	3 (4.1)
Scissors	1 (1.4)

**Table 5:** Injury classified on the basis of the velocity of the projectile N - number of patients

Velocity	N (%)
High velocity	31 (42.47)
Low velocity	41 (56.16)
Combined	1 (1.37)

**Table 6:** Delay in presentation to the emergency department N - number of patients

Time delay	N (%)
> 1 hour	39 (53.42)
< 1 hour	34 (46.58)

**Table 7:** Glasgow Coma Score at presentation N - number of patients

Glasgow Coma Score category	N (%)
Mild derangement (14-15)	68 (93.15)
Moderate derangement (9-13)	0
Severe derangement (3-8)	5 (6.85)

**Table 8:** Abdominal organ evisceration patterns N - number of patients

Abdominal organ eviscerated	N (%)
None	59 (80.8)
Bowel	1 (1.4)
Omentum	12 (16.4)
Bowel and omentum	1 (1.4)

**Table 9:** Co-morbid conditions N - number of patients

Co-morbid condition	N (%)
Diabetes	5 (6.8)
Asthma	1 (1.4)
Hypertension	2 (2.7)
Cardiomyopathy	1 (1.4)
IV drug abuse and dependence	3 (4.1)
Psychiatric illness	4 (5.5)
Tuberculosis	1 (1.4)

**Table 10:** Chest radiograph findings N - number of patients who had a radiograph and had the specific finding

Radiographic abnormality	N (%)
Cardio-pulmonary angle blunting	2 (3.6)
Pneumothorax	1 (1.8)
Air under the diaphragm	6 (10.9)
Pneumothorax	2 (3.6)

**Table 11:** Computed tomography scan findings CT - computed tomography; N - number of patients who underwent the CT scanning and had the specified finding

CT scan abnormality	N (%)
Lung injury	12 (27.9)
Diaphragm injury	4 (9.3)
Intra-abdominal solid organ injury	15 (34.9)
Pneumoperitoneum or hollow viscus injury	11 (25.6)
Bone injury	5 (11.6)
Head injury	2 (4.7)

**Table 12:** Surgical intervention in patients N - number of patients

Surgical intervention		N (%)
Yes	Damage control surgery	4 (5.47)
	Definitive surgery	44 (60.27)
No		25 (34.25)

**Table 13:** Organs injured N - number of patients

Organ	N (%)
Small bowel	19 (26)
Liver	14 (20.3)
Spleen	6 (8.2)
Stomach	9 (12.3)
Pancreas	1 (1.4)
Kidney	7 (9.6)
Colon and rectum	16 (21.9)
Duodenum	3 (4.1)
Diaphragm	10 (13.7)
Abdominal vascular injury	22 (30.1)
Gall bladder	1 (1.4)
Penis	1 (1.4)
Urinary bladder	2 (2.7)
Urethra	1 (1.4)
Pelvic bone	2 (2.7)

**Table 14:** American Association for the Surgery of Trauma - Organ Injury Scale (AAST-OIS) classification of the liver, spleen and kidney AAST-OIS - American Association for the Surgery of Trauma-Organ Injury Scale; N - number of patients

Organ	AAST-OIS grade I - N (%)	AAST-OIS grade II - N (%)	AAST-OIS grade III - N (%)	AAST-OIS grade IV - N (%)	AAST-OIS grade V - N (%)
Liver	0	0	8 (57.1)	6 42.9)	0
Spleen	0	1 (16.7)	2 (33.3)	2 (33.3)	1 (16.7)
Kidney	0	0	5 (71.4)	1 (14.3)	1 (14.3)

**Table 15:** Mortality and complication rates N - number of patients

Outcome	Complications	N (%)
Survived	Yes	37 (50.68)
	No	31 (42.46)
Deceased		5 (6.8)

**Table 16:** Intensive treatment unit admission ITU - intensive treatment unit; N - number of patients

Duration of ITU admission	N (%)
< 1 day	7 (9.5)
1 day – 7 days	5 (6.8)
> 7 days	1 (1.4)

**Table 17:** Complications observed N - number of patients

Complication	N (%)
Contrast-induced compartment syndrome	1 (1.4)
Stoma	7 (9.6)
Pneumonia	9 (12.3)
Acute respiratory distress syndrome	1 (1.4)
Pyothorax	1 (1.4)
Renal failure	1 (1.4)
Surgical site infection	22 (30.1)
Pancreatic fistula	1 (1.4)
Vesico-enteric fistula	2 (2.7)
Diaphragm repair failure	1 (1.4)
Leakage from bowel anastomosis/repair	2 (2.7)

## Discussion

We were able to report on the pattern and characteristics of penetrating abdominal trauma presentations, initial investigations and outcomes, including complications and 30-day mortality, from a major trauma centre in a metropolitan city in India.

The majority of the injuries were due to interpersonal violence, of which 47.9% were due to stab wounds, followed closely by gunshot wounds. The patients were predominantly male and the mean age was 28.19 years. PAT contributed to 3.9% of trauma admissions. In other Indian case series with rural demographics, the mean age, sex predilection, major cause and mode of injury were more or less similar to our data. However, the incidence of bull-gore injuries was high in the other two Indian studies, at 25% and gunshot wounds were starkly low [11,16]. This could be because agriculture is the predominant occupation in those demographics as compared to city dwellers in our study. Unlicensed country-made firearms were a major modality, accounting for 38.4% of the injuries. Access to unlicensed country-made firearms is a festering problem in metropolitan cities of developing nations, with other reports attributing the high rate of homicidal fatal firearm injuries to this particular reason [17]. There were three patients who sustained an injury from a police rifle knife. This is quite a rare occurrence in comparison with data nationally and internationally.

The Western European data show a low percentage of abdominal stab wounds [3]. The demographics and the mean age of the patients from developed countries were similar to our data [5,18]. In 2022, in the series by Saar et al., though the demographics were reflective, the incidence of stab wounds was 95.8% and that of gunshot wounds was 4.2% [19]. A high incidence of gunshot wounds was reported in another series by Shah et al., which was from a low- and middle-income country demographic [20]. There is no consistency between gunshot wounds and stab wounds and cannot be correlated to demographics or geography.

Workplace injuries continue to cause a sizeable percentage of penetrating abdominal trauma. This could be due to accidental sharp force objects or machinery. Yelin et al., in 2016, reported 25% of injuries to be due to one of the two in their case series [21]. Accidental sharp force injuries were further analysed in another case series, with 2.5% attributability [10]. In our series, though we recorded the objects causing injury, we did not delineate workplace and non-workplace injuries.

The management of penetrating abdominal trauma depends on the peritoneal perforation, involvement of organs and hemodynamic status. The bowel and omentum are the most common organs to be eviscerated through the entry wound [18,22]. In our case series, the majority did not have any eviscerations. Amongst the eviscerations, the omentum was the most common organ to be eviscerated.

The small bowel was the most common intra-abdominal organ to be injured in our series. Abdominal vascular injuries as a whole had a higher rate, but we considered the small bowel to be the indicative metric for the highest number, as this was a defined intra-abdominal organ. This was similar to other PAT series reports [6,8,16,18,19,22]. This is because of the attachment to the mesentery, which restricts mobility and increases vulnerability [19]. The outliers to this pattern were the patients of Shah AA et al., who in their series in 2015, reported the colon to be the most commonly injured organ and the liver was reported in another series [11,20].

Peritoneal penetration is the best indicator for laparotomy with a high sensitivity and positive predictive value [16,22]. In our series, we could observe a peritoneal breach in more than half the number of patients, which was detected using clinical examination, ultrasonogram or computed tomography scanning.

After the initial assessment, 75.3% had initial radiographs and 20% of them showed significant findings. Air under the diaphragm was the most common finding in these patients, which is very similar to another case series [22].

The importance of eFAST (extended Focused Abdominal Sonography for Trauma) and CT scanning in patients with penetrating abdominal trauma has been highlighted in the literature [23-25]. eFAST is an intervention that can be performed rapidly in the emergency room and screens the subphrenic, subhepatic, paracolic gutters, pelvis and pericardium for the presence of fluid. The limitations are that it cannot ascertain the origins of the fluid or detect perforations [26]. Sixty patients (82.19%) underwent an eFAST scan and 52.1% were found to have trauma-induced fluid in the abdomen.

The CT scan in the emergency department is used in hemodynamically stable patients, to detect peritoneal violations, free intra-peritoneal fluid or air, injury to a hollow or solid organ, suspected diaphragm injury or wound trajectories clearly penetrating the abdominal cavity but not sensitive for bowel injuries [18,27]. Ninety point four one per cent (90.41%) of the patients underwent a CT scan in our series and 27.9% had abnormalities. Intra-abdominal solid organ injury was the most common abnormality detected by CT scanning in our series.

On initial assessment, 93.15% of our patients had mild (14-15) derangement of the Glasgow Coma Scale (GCS). The mean GCS of these patients was 14.48 (SD=1.96). Other studies align with this observation, reporting only a slight alteration in the GCS of the majority of PAT patients [18,20,28].

The Selective Non-Operative Management (SNOM) protocol for penetrating abdominal trauma published by Shaftan et al. almost 60 years ago and the validated Western German Trauma Association algorithm have reduced unnecessary laparotomies [18,29-31]. Patients without peritonitis, evisceration and hemodynamic shock are triaged to be treated non-operatively by this algorithm [32,33]. Early laparotomy as damage control surgery is indicated in hemodynamically unstable patients with a re-look procedure if required in 48 hours [34]. Five point four seven per cent (5.47%) of the patients underwent surgery in the first 48 hours by this algorithm in our series, with 60.27% requiring definitive surgery at a later date. In the series by Saar et al., 43.7% of the patients underwent immediate laparotomy [19]. Other Indian studies have reported more than 50% of the patients undergoing immediate laparotomy [11,16]. This could be associated with the increased bull-gore injuries reported by both Indian authors, which are associated with higher rates of peritoneal perforation and evisceration. Stringent implementation of the SNOM and the Western German Trauma Association algorithm for PAT could have also led to the lower laparotomies in our series.

We recorded 30-day outcomes in terms of length of ITU stay, complications and mortality. The mortality rate was 7.8% and the morbidity rate was 50.7% among the survivors. The majority of ITU admissions were for one day or less and the average length of hospital stay was 9.75 days (SD=9.16). In the South African and British health systems, the mortality rate for this particular injury was below 3% [5,35]. In Germany, Malkommes reported a mortality of 1.7% and morbidity of 8.7% [18]. This was from 10-year registry data as compared to our data, which is from 18 months. The time spread and a more effective healthcare system could have contributed to this. In a series of abdominal gunshot wounds alone, the mortality rate was 34.3% [20]. Firearm injuries have higher mortality rates due to the explosive nature of the projectile and the projectile's ballistic in vivo, which could explain this. Other studies have reported mortality rates of 15%, which is closer to the mortality in our series. In a series by Shah KD et al., the mortality rate dropped to 2%, which is an outlier considering that the period of the study and the demographic were similar to other studies, including our study, reporting higher rates. The average stay in the hospital ranged from seven to nine days [16,19,20,22]. The observations from our cohort fall well within this range.

The most common cause of complications and morbidity in our series was surgical site infections (30.1%). Siddharth et al. reported wound dehiscence as the most common complication as did Shah KD et al., along with wound infection [16,22]. These studies were carried out in similar healthcare systems, reflecting the same major type of complication.

The strengths of this study are that it is the first to analyse penetrating abdominal trauma presentations and outcomes from New Delhi, the national capital of India. The AAST-OIS classification system has been used, which has seldom been used in similar studies.

The drawbacks are that it is a single-centre study done over a limited time window of 18 months. The classification of these injuries as workplace and non-workplace would have helped us understand the need for industrial safety better.

## Conclusions

This study presents an analysis of the patterns, causes, assessment parameters, investigations, interventions and outcomes of abdominal penetrating trauma from a major trauma centre in a busy metropolitan city. Stab wounds from interpersonal violence in the third and fourth decades form the major chunk of our series. The small bowels are commonly injured and wound-related complications are the most common occurrences. The application of a validated management algorithm, emphasizing the non-operative management of selected patients, can be beneficial to the patient and the health service.
